# High Prevalence and Risk Factors for Infection with Human T-Lymphotropic Viruses 1 and 2 in the Municipality of Ananindeua, Pará, Northern Brazil

**DOI:** 10.3390/v17060765

**Published:** 2025-05-28

**Authors:** Dara da Costa Soares, Felipe Teixeira Lopes, Gabriel dos Santos Pereira Neto, Luciana Cristina Coelho Pantoja Santos, Aline Cecy Rocha Lima, Bruno Sarmento Botelho, Isabella Nogueira Abreu, Felipe Bonfim Freitas, Ricardo Ishak, Izaura Maria Vieira Cayres Vallinoto, Antonio Carlos Rosário Vallinoto

**Affiliations:** 1 Laboratory of Virology, Institute of Biological Sciences, Federal University of Pará, Belém 66075-110, Pará, Brazil; daracsoares@gmail.com (D.d.C.S.); feliptlopes1@gmail.com (F.T.L.); gabrielnetoenf@gmail.com (G.d.S.P.N.); lccp.santos27@gmail.com (L.C.C.P.S.); alinececy@yahoo.com (A.C.R.L.); bruno.botelho@ics.ufpa.br (B.S.B.); isabella.nogueira@oulook.com.br (I.N.A.); rishak@ufpa.br (R.I.); ivallinoto@ufpa.br (I.M.V.C.V.); 2 Virology Section, Evandro Chagas Institute, Ananindeua 67030-000, Pará, Brazil; felipebonfim@iec.gov.br

**Keywords:** HTLV-1, HTLV-2, epidemiology, Amazonia, Ananindeua, Pará

## Abstract

This descriptive, observational, cross-sectional study evaluated HTLV-1 and HTLV-2 infections in Ananindeua, northern Brazil. Individuals were screened for anti-HTLV-1/2 using ELISA (Murex HTLV-I + II, DiaSorin). Reactive or indeterminate samples underwent confirmation via Western blot (HTLV Blot 2.4 kit, MP Diagnostics) and/or RT-qPCR. A questionnaire examined behavioral and risk factors for HTLV-1/2 infection. HTLV-positive individuals received counseling, nurse follow-up, and specialized medical care. Among the 228 individuals investigated, 6 (2.7%) were infected with HTLV-1: 4 men (66.67%) and 2 women (33.33%), aged 51–73 years. The only significant risk factor observed was blood transfusion. Additionally, 80 other individuals residing in the municipality of Ananindeua independently visited the laboratory for an HTLV-1/2 diagnosis. Among them, 23 were diagnosed with HTLV-1 infection, and 1 with HTLV-2. Among the 30 positive individuals, 80% were asymptomatic, while 20% exhibited clinical manifestations associated with HTLV infection, including HAM and Sézary syndrome. These results indicate a notable prevalence of HTLV-1 infection in the municipality of Ananindeua emphasizing the significance of diagnosing the infection to assess its prevalence across the country accurately.

## 1. Introduction

Human T-lymphotropic viruses 1 and 2 (HTLV-1 and HTLV-2) were described as the first human oncogenic retroviruses, isolated in the 1980s from patients with cutaneous T-cell lymphoma and a T-cell variant of hairy cell leukemia, respectively [1,2]. The viruses present tropism for CD4+ and CD8+ T cells and are currently associated with adult T-cell leukemia/lymphoma (ATLL) and inflammatory diseases like HTLV-1-associated myelopathy (HAM), uveitis, infective dermatitis, arthritis, and other illnesses [3,4,5,6,7,8,9]. HTLV-1/2 are transmitted by direct contact with contaminated bodily fluids, and the main transmission routes are blood transfusion, sharing of contaminated syringes and needles, sexual intercourse without condoms, and mother-to-child transmission during pregnancy, childbirth, and, mainly, breastfeeding [5,10,11,12,13,14].

HTLV-1/2 are neglected and endemic infections present in different regions of the world, with the highest prevalences observed in Southern Japan, the Americas, Africa, and the Caribbean. The number of infected people worldwide is underestimated, but the global estimates range from 5 million to 10 million people living with HTLV-1/2 (PLHTLV) worldwide [5,14,15].

The origin of HTLV occurred through human interaction with simian T-lymphotropic viruses 1 and 2 (STLV-1/2), and the viruses spread throughout the world approximately 100,000 years ago due to human migration from Africa to other continents, reaching the Americas through the Bering Strait [16,17,18].

The introduction of HTLV-1 in Brazil was probably due to the human migration of African people during the slave trade, and HTLV-2 by the ancient human migration from Asia [18]. Brazil is considered one of the endemic countries for HTLV-1/2 infection, with an estimated 800,000 to 2.5 million people infected [19]. Additionally, epidemiological data demonstrated a high prevalence rate of infected people in some states of the northern and northeastern regions of Brazil, such as Bahia, Maranhão, and Pará [13,20,21].

The State of Pará is one of the federative units of Brazil with the highest prevalence of HTLV-1/2 infection, with the viruses disseminated among several population groups, such as *quilombolas*, pregnant women, blood donors, and indigenous people [18,20,21,22,23,24]. It is important to highlight that studies conducted in the urban region describe the circulation of both HTLV-1 and HTLV-2 in the state capital, Belém [25,26,27]. Nevertheless, there are several groups without any information on their epidemiological statuses regarding the presence of HTLV-1/2 in this region.

This study aimed to evaluate the serological and molecular evidence of HTLV-1 and HTLV-2 infections among residents of Ananindeua, Pará. This city is part of the metropolitan area of Belém and is the second most populous municipality in the state, playing a significant role due to its economy and geographic location.

## 2. Materials and Methods

### 2.1. Study Design and Population

This is a descriptive, observational, cross-sectional study that occurred from 2020 to 2024. The first approach occurred from 2020 to 2021, aiming to evaluate the prevalence of HTLV-1/2 infection in residents from Ananindeua. Serological screening and confirmatory tests were performed through an active search for people infected with HTLV-1/2, and the participants were selected through campaigns in community centers, churches, and public places. During this period, a total of 228 randomly selected individuals from various age groups and both genders were tested for HTLV-1/2 infection.

From 2022 onwards, with the consolidation of the Service for Assistance to People Living with HTLV (SAPEVH), and considering the number of seropositive individuals in the first sampling, a second effort was launched to increase the number of people investigated among the population of Ananindeua, selecting residents of that municipality who spontaneously sought the SAPEVH in the period between 2022 and 2024, seeking a diagnosis for HTLV-1/2 infection.

All participants were informed about the objective, risks, and benefits of this study and gave their consent by signing Informed Consent forms. Individuals of both sexes were randomly selected to join the present study by spontaneous demand and were submitted to a questionnaire investigating behavioral and risk factors for HTLV-1/2 infection. The SAPEVH aimed to provide diagnosis for the general public, and a follow-up for those who were seropositive for HTLV-1/2, including serological screening, confirmatory tests, and, for seropositive patients, counseling and follow-up with a nurse and specialized medical assistance. From 2022 to 2024, a total of 80 people were assisted by this service.

This study was approved by the Human Research Ethics Committee of the Health Sciences Institute of the Federal University of Pará and the National Research Ethics Committee (CONEP), in compliance with fundamental ethical and scientific requirements, respecting Resolution N° 466/12 of the Ministry of Health, which regulates research involving human beings under number CAAE: 27290619.2.0000.0018 and opinion 4.351.470.

### 2.2. Serological Tests

A peripheral venous blood sample (4.5 mL) was obtained and screened for anti-HTLV-1/2 by ELISA (Murex HTLV-I + II, DiaSorin, Dartford, UK) according to the manufacturer’s instructions. Samples considered reactive or indeterminate were submitted to confirmatory tests using Western blot (HTLV Blot 2.4 kit, MP Diagnostics, Singapore, Republic of Singapore) and/or Real-Time Polymerase Chain Reaction (RT-qPCR).

### 2.3. DNA Extraction

Seropositive samples were submitted to DNA extraction for the detection of HTLV proviral DNA. DNA was extracted from 200 µL of leukocytes using the QIAamp DNA mini kit (Qiagen, Hilden, Germany) following the manufacturer’s protocol.

### 2.4. Real-Time PCR

The molecular analysis of HTLV infection was conducted via qPCR using the TaqMan system (Applied Biosystems, Foster City, CA, USA) on the Applied Biosystems StepOne Plus Real-Time PCR platform. The albumin gene (141 bp) was used as endogenous control, and the non-homologous viral pol gene regions (186 bp) of HTLV-1 and HTLV-2 were used as molecular markers to identify viral particles [28]. Each reaction contained 12.5 µL of TaqMan Universal PCR Master Mix (2X) (Applied Biosystems, Foster City, CA, USA), 6.0 µL of ultrapure water, 0.5 µL of each primer, 0.5 µL of each probe, and 5.0 µL of DNA, resulting in a total volume of 25 µL. The following temperature cycles were carried out: 95 °C for 10 min, followed by 45 cycles of 95 °C for 15 s, and then 60 °C for binding primers and probes for 1 min. The following primers were used in those reactions: 5′-ccctacaatccaaccagctcag-3′ (HTLV-1F), 5′-gtggtgaagctgccatcgggtttt-3′ (HTLV-1R), 5′-cgattgtgtacaggccgattg-3′ (HTLV-2F), 5′-caggagggcatgtcgatgtag-3′ (HTLV-2R), 5′-gctgtcatctcttgtgggctgt-3′ (AlbuminF), and 5′-aaactcatgggagctgctggtt-3′ (Albumin R). The probe sequences were as follows: FAM-5′-ctttactgacaaacccgacctacccatgga-3′-MGB (HTLV-1), FAM-5′-tgtcccgtctcaggtggtctatgttcca-3′-MGB (HTLV-2), and FAM-5’-cctgtcatgcccacacaaatctc-3′-MGB (Albumin).

### 2.5. Statistical Analysis

Data obtained from questionnaire responses, including population characteristics, risk factors, and demographic data, were tabulated in Microsoft Office Excel and then analyzed and described using descriptive statistical analysis. The risk factors associated with HTLV-1/2 infection were analyzed by Pearson’s chi-square test and G test, considering a level of 5% (*p*-value < 0.05). Data analysis was performed by the software BioEstat version 5.0 [29].

## 3. Results

### 3.1. Serological Screening, Molecular Analyses, and Prevalence

In the first approach of the present study, 228 individuals were enrolled in the investigation, of whom 39.47% were males and 60.53% were females. The screening serology test showed six individuals seropositive for HTLV-1 (2.7%), who were then submitted for molecular confirmation by qPCR. All samples were confirmed for HTLV-1 infection, of which four were males (66%) and two were females (33%), with ages ranging from 51 to 73 years old (Table 1).

The epidemiological characteristics of the 228 individuals indicated that 55.70% self-identified as mixed race, 20.18% reported having a secondary education level, and 29.82% declared having an income of one to two minimum wages. There was no significant difference between the infected and non-infected groups.

Most HTLV-1 patients received a family income higher than the minimum wage, 16.67% reported a family income less than or equal to the minimum wage, 50% reported living with three to four minimum wages, and 16.67% declared receiving more than five minimum wages. Regarding the educational situation, there was a predominance of a high school educational level: 33.33% declared to have completed elementary school, 50% finished high school, and 16.67% reported an unfinished university degree (Table 1).

Between 2022 and 2024, a total of 80 individuals living in the municipality of Ananindeua voluntarily sought the SAPEVH for a diagnosis of HTLV-1/2 infection, comprising 25 males and 55 females. Their ages ranged from 13 to 84 years old. Twenty-four subjects tested positive for HTLV-1/2 in the screening, and 20 were confirmed by RT-qPCR. Of the four remaining samples, one showed amplification of proviral DNA for the HTLV-2 pol-II region and three were undetectable by RT-qPCR. However, when subjected to Western blot as an alternative for diagnosis, the three samples showed reactivity for HTLV-1. Of the 24 HTLV-1/2-positive patients who attended the SAPEVH in the period from 2022 to 2024, 14 were female (58.33%) and 10 were male (41.67%), and their ages ranged from 19 to 72 years old.

### 3.2. Clinical Findings

After the confirmatory laboratory diagnosis, patients were evaluated by a multidisciplinary health team in search of clinical manifestations of diseases associated with HTLV-1/2. Of the 30 positive individuals, 80% were asymptomatic, and 20% presented clinical manifestations associated with HTLV infection. Patients #7, #8, and #22 presented systemic lupus erythematosus, fibromyalgia, and motor deficit, respectively. Patients #13 and #20 were clinically diagnosed with HAM, while patient #23 presented Sézary syndrome (Table 2).

### 3.3. Epidemiological Characteristics and Risk Factors Associated with HTLV-1/2 Infection

The most significant risk factor associated with HTLV-1/2 infection was blood transfusion (*p* = 0.0028; odds ratio = 4.18; IC95%: 1.73–10.11; *p* < 0.05), but it was not related to having received a transfusion before 1993 (*p* = 0.3431), when mandatory testing for HTLV-1/2 was implemented in Brazilian Blood Centers. However, other important risk factors were also reported by infected individuals (without statistical significance), such as unprotected sexual intercourse, breastfeeding during childhood, the presence of tattoos or piercings, the use of illicit drugs, having sex for money, and a previous diagnosis of another STI (Table 3).

**Figure 1:**
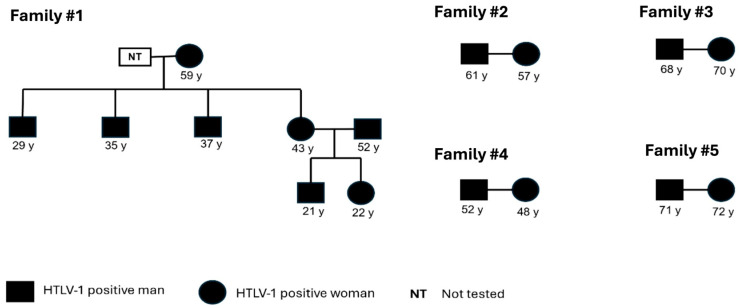
Pedigree showing possible intrafamilial transmission routes. All families present horizontal transmission, and Family #1 presents both vertical and horizontal transmission.

During the course of the investigation, it was possible to pinpoint other family members who were included in the present study for screening and confirmation. Family #1 was identified with eight members that tested positive for HTLV-1; the index case was a man (37 y). Family clusters were detected among four other families (#2, #3, #4, and #5) with the occurrence of infection among couples, suggesting that sexual intercourse was the main route of transmission. Unfortunately, it was not possible to detect infection among the children of these couples. (Figure 1). Furthermore, intrafamilial transmission could not be investigated for the only individual infected with HTLV-2.

## 4. Discussion

The present study reported a prevalence of 2.7% HTLV-1 infection in the urban population of Ananindeua, Pará, Brazil. As far as we know, despite the small sample size studied, this is the highest prevalence of HTLV-1 infection in an urban population in Brazil, surpassing the figure (1.18%) observed in Salvador [30].

This study represents the first epidemiological investigation of HTLV infection in the population residing in the municipality of Ananindeua. Silva et al. [31] reported the prevalence of HTLV infection, as high as 2%, in the metropolitan region of the capital city, Belém, but it is important to mention that the sample included individuals residing in Ananindeua. In contrast, in our previous study, conducted only with the population of Belém [25], the prevalence found was only 0.38%. Differences in the study population, despite geographical proximity and a shared economic context, may account for the disparity in prevalence rates.

The Ananindeua municipality originated from the quilombo community of Abacatal, which was initially formed by escaped African slaves. This community dates back to the 18th century and played a significant role in the formation of Ananindeua [32,33]. During the colonial period, the forced human migration was clearly increased from several parts of the African continent, and people were brought to Brazil as slaves, particularly to important ports, including Rio de Janeiro, Salvador, Recife, São Luiz, and Belem [18,34]. The high prevalence of HTLV-1 in Ananindeua may likely be related to the African origin of the virus and its introduction to Brazil during the slave trade in the colonial period.

Of the 30 individuals infected with HTLV-1/2 analyzed in this study, the majority were females, a finding that agrees with previous reports, in which women were found to be more affected by HTLV-1/2 infection than men [24,35,36,37,38,39]. The questionnaires revealed that females over 50 years old were the most affected group in this study. These findings are consistent with previous studies, which identified a correlation between age and the increase in prevalence rates for HTLV infection [14,40]. The association between age and sex may be due to the increased likelihood of acquiring the virus over time, as well as the fact that the virus is more efficiently transmitted from men to women. Kaplan et al. [41] hypothesized that the virus is more efficiently transmitted in a male-to-female manner due to the higher viral load present in men’s semen. Other studies have suggested that this difference in transmission could be related to the higher number of lymphocytes found in semen compared to vaginal secretions [42].

The main risk factors associated with seropositivity to HTLV-1/2, included the practice of unprotected sex, breastfeeding, and low income, although those were not considered statistically significant. Nevertheless, the presence of substantial evidence indicating intrafamilial transmission suggests that unprotected sexual activity may play a significant role in this mode of transmission. The sexual transmission of HTLV represents an important risk factor for the spread of the virus. There is substantial evidence showing the importance of this epidemiological transmission within close communities such as Indigenous people in Brazil [18,24,43,44], as well as among urban communities in the Amazon region of Brazil [45]. A study conducted in Salvador, Brazil, has shown that the sexual transmission route remains the most important mechanism of transmission, showing the increase in the prevalence in older age groups of infected people, which supports the idea of the acquisition of the virus over time [46].

In the case of family #1, we hypothesized that breastfeeding was the main transmission route, as the mother reported breastfeeding for 6 months or more. Long-term breastfeeding and the high proviral load present in breast milk are known risk factors for HTLV transmission [47]. It is recommended that HTLV-1/2-positive mothers refrain from breastfeeding, as there is evidence showing that the duration of breastfeeding (up to 6 months) is associated with an increased risk of transmission [13,48]. However, this recommendation does not fully reflect the situation in developing countries, where breast milk often serves as the primary source of nutrition for infants during their first 2 years. In this context, poverty and the lack of public health policies underscore the need for further studies and the development of effective strategies to prevent HTLV transmission from mother to child through breastfeeding [49].

Our results showed, for the first time, a high prevalence of HTLV-1 infection in Ananindeua municipality, which, to our knowledge, is the highest-ever reported prevalence for an urban population in Brazil. These findings highlight the urgent need for ongoing epidemiological surveillance in Ananindeua and other regions of Brazil with a strong mixture of African descendants where HTLV-1/2 prevalence has not been investigated. They also emphasize the importance of promoting services that offer diagnostic and confirmatory tests for individuals living with HTLV-1/2 to gain a clearer understanding of the true prevalence across the country.

## Figures and Tables

**Table 1:** General characteristics of the study population.

Variables	Total *n* (%)	Positive *n* (%)	Negative *n* (%)	*p*-Values	IC95%
**Prevalence**	228 (100)	6 (2.7)	222 (97.3)	-	0.6–4.7%
**Sex**					
Male	90 (39.47)	4 (66.67)	86 (38.74)	0.2160	-
Female	138 (60.53)	2 (33.33)	136 (61.26)		
**Ethnicity**					
White	54 (23.68)	0	54 (24.32)	0.4639	-
Black	36 (15.79)	1 (16.67)	35 (15.77)		
Mixed	127 (55.70)	5 (83.33)	122 (54.95)		
Asian	5 (2.20)	0	5 (2.25)		
Non informed	6 (2.63)	0	6 (2.70)		
**Age (years)**					
6 to 11	7 (3.07)	0	7 (3.15)	0.0925	-
12 to 17	12 (5.26)	0	12 (5.41)		
18 to 29	79 (34.65)	0	79 (35.59)		
30 to 59	90 (39.47)	2 (33.33)	88 (39.64)		
≥60	34 (14.91)	4 (66.67)	30 (13.51)		
Non informed	6 (2.63)	0	6 (2.70)		
**Education level**					
Illiterate	0	0	0	1	-
Elementary (Unfinished)	17 (7)	0	17 (8)		
Elementary	13 (5.70)	2 (33.33)	11 (4.95)		
High school (Unfinished)	12 (5.26)	0	12 (5.41)		
High school	46 (20.18)	3 (50)	43 (19.37)		
College (Unfinished)	47 (20.61)	1 (16.67)	46 (20.72)		
College	29 (12.72)	0	29 (13.06)		
Postgraduate (Unfinished)	19 (8.33)	0	19 (8.56)		
Postgraduate	44 (19.30)	0	44 (19.82)		
Non informed	1 (0.44)	0	1 (0.45)		
**Income (minimum wages)**					
<1	23 (10.09)	1 (16.67)	22 (9.91)	0.6588	-
1 to 2	68 (29.82)	1 (16.67)	67 (30.18)		
3 to 4	60 (26.32)	3 (50.00)	57 (25.68)		
≥5	57 (25.00)	1 (16.67)	56 (25.23)		
Non informed	20 (8.77)	0	20 (9)		

**Table 2:** Characteristics of individuals residing in Ananindeua who spontaneously sought diagnostic services through the SAPEVH.

Collection Source	Patient	Age	Sex	CM	ELISA	qPCR	Western Blot	Result
Active search	#01	54	F	A	+	Detectable		HTLV-1
	#02	61	M	A	+	Detectable		HTLV-1
	#03	64	F	A	+	Detectable		HTLV-1
	#04	70	M	A	+	Detectable		HTLV-1
	#05	64	M	A	+	Detectable		HTLV-1
	#06	50	M	A	+	Detectable		HTLV-1
SAPEVH	#07	44	F	Lupus	+	Detectable		HTLV-1
	#08	34	F	Fibromyalgia	+	ND	HTLV-1	HTLV-1
	#09	56	M	A	+	Detectable		HTLV-1
	#10	40	M	A	+	Detectable		HTLV-2
	#11	64	F	A	+	Detectable		HTLV-1
	#12	37	M	A	+	Detectable		HTLV-1
	#13	33	M	HAM	+	Detectable		HTLV-1
	#14	42	F	A	+	Detectable		HTLV-1
	#15	40	F	A	+	Detectable		HTLV-1
	#16	52	M	A	+	Detectable		HTLV-1
	#17	19	M	A	+	ND	HTLV-1	HTLV-1
	#18	58	F	A	+	Detectable		HTLV-1
	#19	40	F	A	+	Detectable		HTLV-1
	#20	52	M	HAM	+	Detectable		HTLV-1
	#21	48	F	A	+	ND	HTLV-1	HTLV-1
	#22	59	F	Motor Deficit	+	Detectable		HTLV-1
	#23	71	M	SS	+	Detectable		HTLV-1
	#24	45	M	A	+	Detectable		HTLV-1
	#25	64	F	A	+	Detectable		HTLV-1
	#26	71	F	A	+	Detectable		HTLV-1
	#27	29	M	A	+	Detectable		HTLV-1
	#28	22	F	A	+	Detectable		HTLV-1
	#29	72	F	A	+	Detectable		HTLV-1
	#30	48	F	A	+	Detectable		HTLV-1

CM: Clinical manifestation; ND: not detectable; F: female; M: male; A: asymptomatic; HAM: HTLV-1-associated myelopathy; SS: Sézary syndrome.

**Table 3:** Risk factors associated with HTLV infection.

Risk Factors	Total N (%)	Positive N (%)	Negative N (%)	*p*-Values
**Tattoo**				
Yes	44 (14.29)	5 (16.67)	39 (14.03)	0.7826
No	260 (84.42)	25 (83.33)	235 (84.53)	
Not reported	4 (1.30)	0 (0)	4 (1.44)	
**Piercing**				
Yes	24 (7.79)	3 (10.00)	21 (7.55)	0.7149
No	265 (86.04)	27 (90.00)	238 (85.61)	
Not reported	19 (6.17)	0 (0)	19 (6.83)	
**Blood transfusion**				
Yes	34 (11.04)	9 (30.00)	25 (8.99)	0.0028 **
No	265 (86.04)	21 (70.00)	244 (87.77)	
Not reported	9 (2.92)	0 (0)	9 (3.24)	
**Blood transfusion period**				
Before 1993	9 (26.47)	3 (33.33)	6 (24.00)	0.3431
After 1993	21 (61.76)	4 (44.44)	17 (68.00)	
Not reported	4 (11.77)	2 (22.22)	2 (8.00)	
**Use of illicit drugs**				
Yes	18 (5.84)	4 (13.33)	14 (5.04)	0.0806
No	275 (89.29)	24 (80.00)	251 (90.29)	
Not reported	15 (4.87)	2 (6.67)	13 (4.68)	
**Sexually active**				
Yes	194 (62.99)	17 (56.67)	177 (63.67)	0.6485
No	88 (28.57)	10 (33.33)	78 (28.06)	
Not reported	26 (8.44)	3 (10.00)	23 (8.27)	
**Use of condoms**				
Yes	106 (34.42)	10 (33.33)	96 (34.53)	0.2827
No	98 (31.82)	15 (50.00)	83 (29.86)	
Sometimes	46 (14.94)	2 (6.67)	44 (15.83)	
Not reported	58 (18.83)	3 (10.00)	55 (19.78)	
**Sex for money**				
Yes	5 (1.30)	1 (3.33)	4 (1.44)	0.3606
No	272 (87.01)	27 (93.33)	245 (86.13)	
Not reported	31 (9.42)	2 (6.67)	29 (10.43)	
**Diagnosis of STI**				
Yes	27 (8.77)	5 (16.67)	22 (7.91)	0.1574
No	232 (75.32)	20 (66.67)	212 (76.25)	
Does not know	20 (6.49)	2 (6.67)	18 (6.47)	
Not reported	29 (9.42)	3 (10.00)	26 (9.35)	

** Odds ratio = 4.18 (IC95%: 1.73–10.11; *p* < 0.05).

## Data Availability

The original contributions presented in this study are included in the article. Further inquiries can be directed to the corresponding author(s).

## References

[1] Poiesz B., Ruscetti F., Gazdar A., Bunn P., Minna J., Gallo R. (1980). Detection and isolation of type C retrovirus particles from Fresh and Cultured Lymphocytes of a patient with cutaneous T-cell lymphoma. Proc. Natl. Acad. Sci. USA.

[2] Kalyanaraman V.S., Sarngadharan M.G., Robert-Guroff M., Miyoshi I., Blayney D., Golde D., Gallo R.C. (1982). A new subtype of human T-cell leukemia virus (HTLV-II) associated with a T-cell variant of hairy cell leukemia. Science.

[3] Gessain A., Barin F., Vernant J., Gout O., Maurs L., Calender A., De Thé G. (1985). Antibodies to human t-lymphotropic virus type-I in patients with tropical spastic paraparesis. Lancet.

[4] Schierhout G., McGregor S., Gessain A., Einsiedel L., Martinello M., Kaldor J. (2020). Association between HTLV-1 infection and adverse health outcomes: A systematic review and meta-analysis of epidemiological studies. Lancet Infect. Dis..

[5] Legrand N., McGregor S., Bull R., Bajis S., Valencia B.M., Ronnachit A., Einsiedel L., Gessain A., Kaldor J., Martinello M. (2022). Clinical and public health implications of human T-lymphotropic virus type 1 infection. Clin. Microbiol. Rev..

[6] Kamoi K. (2020). HTLV-1 in Ophthalmology. Front. Microbiol..

[7] Eguchi K., Iwanaga M., Terada K., Aramaki T., Tuji Y., Kurushima S., Kojima K., Arima K., Iwamoto N., Ichinose K. (2020). Clinical features and human T-cell leukemia virus type-1 (HTLV-1) proviral load in HTLV-1-positive patients with rheumatoid arthritis: Baseline data in a single center cohort study. Mod. Rheumatol..

[8] Martin F., Taylor G.P., Jacobson S. (2014). Inflammatory manifestations of HTLV-1 and their therapeutic options. Expert Rev. Clin. Immunol..

[9] Brites C., Grassi M.F., Quaresma J.A.S., Ishak R., Vallinoto A.C.R. (2021). Pathogenesis of HTLV-1 infection and progression biomarkers: An Overview. Braz. J. Infect. Dis..

[10] Gotuzzo E., Arango C., de Queiroz-Campos A., Istúriz R.E. (2000). Human T-cell lymphotropic virus-I in Latin America. Infect. Dis. Clin. N. Am..

[11] Gonçalves D.U., Proietti F.A., Ribas J.G.R., Araújo M.G., Pinheiro S.R., Guedes A.C., Carneiro-Proietti A.B.F. (2010). Epidemiology, treatment, and prevention of human T-cell leukemia virus type 1-associated diseases. Clin. Microbiol. Rev..

[12] Eusebio-Ponce E., Anguita E., Paulino-Ramirez R., Candel F.J. (2019). HTLV-1 Infection: An emerging risk. pathogenesis, epidemiology, diagnosis and associated diseases. Rev. Esp. Quimioter..

[13] Rosadas C., Menezes M.L.B., Galvão-Castro B., Assone T., Miranda A.E., Aragón M.G., Caterino-De-Araujo A., Taylor G.P., Ishak R. (2021). Blocking HTLV-1/2 Silent Transmission in Brazil: Current public health policies and proposal for additional strategies. PloS Negl. Trop. Dis..

[14] Gessain A., Mahieux R. (2012). Tropical spastic paraparesis and HTLV-1 associated myelopathy: Clinical, epidemiological, virological and therapeutic aspects. Rev. Neurol..

[15] ECDC—European Centre for Disease Prevention and Control (2015). Geographical Distribution of Areas with a High Prevalence of HTLV-1 Infection.

[16] Vallinoto A.C.R., Ishak M.O.G., Azevedo V.N., Vicente A.C.P., Otsuki K., Hall W.W., Ishak R. (2002). Molecular epidemiology of human T-lymphotropic virus type II infection in Amerindian and urban populations of the amazon region of Brazil. Hum. Biol..

[17] Galvão-Castro B., Alcântara L.C., Grassi M.F., Mota-Miranda A.C.A., de Queiroz A.T.L., Rego F.F.A., Mota A.C.A., Pereira S.A., Magalhães T., Tavares-Neto J. (2009). HTLV-I Epidemiology And Origin in Salvador, State of Bahia: The city with the highest prevalence of this infection in Brazil. Gaz. Médic. Bahia.

[18] Ishak R., Ishak M.d.O.G., Azevedo V.N., Machado L.F.A., Vallinoto I.M.C., Queiroz M.A.F., Costa G.d.L.C., Guerreiro J.F., Vallinoto A.C.R. (2020). HTLV in South America: Origins of a silent ancient human infection. Virus Evol..

[19] Carneiro-Proietti A.B.F., Ribas J.G.R., Catalan-Soares B.C., Martins M.L., Brito-Melo G.E.A., Martins-Filho O.A., Pinheiro S.R., Araújo A.d.Q.-C., Galvão-Castro B., de Oliveira M.S.P. (2002). Infecção e doença pelos vírus linfotrópicos humanos de células T (HTLV-I/II) no Brasil. Rev. Soc. Bras. Med. Trop..

[20] Catalan-Soares B., Carneiro-Proietti A.B.d.F., Proietti F.A. (2005). Heterogeneous geographic distribution of human T-cell lymphotropic viruses I and II (HTLV-I/II): Serological screening prevalence rates in blood donors from large urban areas in Brazil. Cad. Saúde Pública.

[21] Rosadas C., Miranda A.E., Gonçalves D.U., Caterino-de-Araujo A., Assone T., Ishak R., Coordenação-Geral de Vigilância das Infecções Sexualmente Transmissíveis (CGIST/DCCI/SVS) (2020). Prevalência da infecção por HTLV-1/2 no Brasil. Bol. Epidemiol. Secr. Vigilânc. Saúde Minist. Saúde.

[22] Vallinoto A., Pontes G., Muto N., Lopes I., Machado L., Azevedo V., Carvalhaes F., Santos S., Guerreiro J., Ishak M. (2006). Identification of human T-cell lymphotropic virus infection in a semi-isolated afro-brazilian quilombo located in the Marajó Island (Pará, Brazil). Mem. Inst. Oswaldo Cruz.

[23] Sequeira C.G., Tamegão-Lopes B.P., Santos E.J.M.D., Ventura A.M.R., Moraes-Pinto M.I., de Menezes Succi R.C. (2012). Descriptive Study of HTLV Infection in a population of pregnant women from the state of Pará, Northern Brazil. Rev. Soc. Bras. Med. Trop..

[24] Abreu I.N., Lima C.N.C., Sacuena E.R.P., Lopes F.T., da Silva Torres M.K., Santos B.C.d., de Oliveira Freitas V., de Figueiredo L.G.C.P., Pereira K.A.S., de Lima A.C.R. (2022). HTLV-1/2 in Indigenous peoples of the Brazilian amazon: Seroprevalence, molecular characterization and sociobehavioral factors related to risk of infection. Viruses.

[25] Lopes F.T., de Sousa R.S., Gomes J.L.C., Vallinoto M.C., de Lima A.C.R., Lima S.S., Freitas F.B., Feitosa R.N.M., da Silva A.N.M.R., Machado L.F.A. (2022). The relevance of a diagnostic and counseling service for people living with HTLV-1/2 in a metropolis of the Brazilian Amazon. Front. Public Health.

[26] Ferreira L.d.S.C., Costa J.H.G., da Costa C.A., Melo M.d.F.C.d., Andrade M.L., Martins L.C., Ishikawa E.A.Y., de Sousa M.S. (2010). Soroprevalência do vírus linfotrópico de células T humanas em comunidades ribeirinhas da região nordeste do estado do Pará, Brasil. Rev. Pan-Amaz. Saúde.

[27] Ishak R., Vallinoto A.C.R., Azevedo V.N., Ishak M.d.O.G. (2003). Epidemiological aspects of retrovirus (HTLV) infection among Indian populations in the amazon region of Brazil. Cad. Saúde Pública.

[28] Tamegão-Lopes B.P., Rezende P.R., Maradei-Pereira L.M.C., de Lemos J.A.R. (2006). Carga proviral do HTLV-1 e HTLV-2: Um método simples através da pcr quantitativa em tempo real [HTLV-1 and HTLV-2 proviral load: A simple method using quantitative Real-Time Pcr]. Rev. Soc. Bras. Med. Trop..

[29] Ayres M., Ayres J., Ayres D., Santos A. Bioestat 5.3: Aplicações Estatísticas nas Áreas das Ciências Biológicas e Médicas.

[30] Pereira F.M., Almeida M.d.C.C.d., Santos F.L.N., Carreiro R.P., Regis-Silva C.G., Galvão-Castro B., Grassi M.F.R. (2019). Evidence of new endemic clusters of human T-cell leukemia virus (HTLV) infection in Bahia, Brazil. Front. Microbiol..

[31] Silva I.C., Pinheiro B.T., Nobre A.F.S., Coelho J.L., Pereira C.C.C., Ferreira L.d.S.C., de Almeida C.P.S., Viana M.d.N.D.S.d.A., de Almeida D.S., Falcão J.R. (2018). Moderate endemicity of the human T-lymphotropic virus infection in the metropolitan region of Belém, Pará, Brazil. Rev. Bras. Epidemiol..

[32] Marin R., Castro E. (1999). No Caminho das Pedras de Abacatal: Experiência Social de Grupos Negros no PARÁ.

[33] Araújo A.d.S., Anjos DRd Silva RdSe Santos MASd Martins C.M., Almeida R.H.C. (2017). Análise socioeconômica de agricultores da comunidade quilombola do Abacatal, Ananindeua, estado do Pará, Brasil. Biota Amaz..

[34] Amoussa A.E.R., Wilkinson E., Giovanetti M., Rego F.F.d.A., Araujo T.H.A., Gonçalves M.d.S., de Oliveira T., Alcantara L.C.J. (2017). HTLV-1aa introduction into Brazil and its association with the trans-Atlantic slave trade. Infect. Genet. Evol..

[35] Vieira B.A., Bidinotto A.B., Dartora W.J., Pedrotti L.G., de Oliveira V.M., Wendland E.M. (2021). Prevalence of Human T-Lymphotropic Virus Type 1 And 2 (HTLV-1/-2) Infection in pregnant women in Brazil: A systematic review and meta-analysis. Sci. Rep..

[36] Alencar S.P., Souza M.d.C., Fonseca R.R.d.S., Menezes C.R., Azevedo V.N., Ribeiro A.L.R., Lima S.S., Laurentino R.V., Barbosa M.d.A.d.A.P., Freitas F.B. (2020). Prevalence and molecular epidemiology of human T-lymphotropic virus (HTLV) infection in people living with HIV/Aids in the Pará state, Amazon region of Brazil. Front. Microbiol..

[37] Dourado I., Alcantara L.C., Barreto M.L., Teixeira M.d.G., Galvão-Castro B. (2003). HTLV-I in the general population of Salvador, Brazil: A city with African ethnic and sociodemographic characteristics. J. Acquir. Immune Defic. Syndr..

[38] de Alcântara Maneschy C., Barile K.A.D.S., de Castro J.A.A., Palmeira M.K., de Castro R.B.H., de Melo Amaral C.E. (2022). Seroprevalence of the human T lymphotropic virus (HTLV 1 AND HTLV 2) in blood donor candidates in the state of Pará, northern Brazil. Res. Soc. Dev..

[39] Miranda C., Utsch-Gonçalves D., Piassi F.C.C., Loureiro P., Gomes I., Ribeiro M.A., de Almeida-Neto C., Blatyta P., Amorim L., Mateos S.O.G. (2022). Prevalence and risk factors for human T-cell lymphotropic virus (HTLV) in blood donors in Brazil—A 10-year study (2007–2016). Front. Med..

[40] Eshima N., Iwata O., Iwata S., Tabata M., Higuchi Y., Matsuishi T., Karukaya S. (2009). Age and gender specific prevalence of HTLV-1. J. Clin. Virol..

[41] Kaplan J.E., Khabbaz R.F., Murphy E.L., Hermansen S., Roberts C., Lal R., Heneine W., Wright D., Matijas L., Thomson R. (1996). Male-to-female transmission of human T-cell lymphotropic virus types I and II: Association with viral load. the retrovirus epidemiology donor study group. J. Acquir. Immune Defic. Syndr. Hum. Retrovirol..

[42] Paiva A., Casseb J. (2014). Sexual transmission of human T-cell lymphotropic virus type 1. Rev. Soc. Bras. Med. Trop..

[43] Ishak R., Harrington W.J., Azevedo V.N., Eiraku N., Ishak M.O., Guerreiro J.F., Santos S.B., Kubo T., Monken C., Alexander S. (1995). Identification of human T cell lymphotropic virus type IIa infection in the Kayapo, an Indigenous population of Brazil. AIDS Res. Hum. Retroviruses.

[44] Martel M., Gotuzzo E. (2022). HTLV-1 is also a sexually transmitted infection. Front. Public Health.

[45] da Costa C.A., Furtado K.C.Y.O., Ferreira L.d.S.C., Almeida D.d.S., Linhares A.d.C., Ishak R., Vallinoto A.C.R., de Lemos J.A.R., Martins L.C., Ishikawa E.A.Y. (2013). Familial transmission of human T-cell lymphotropic virus: Silent dissemination of an emerging but neglected infection. PLoS Negl. Trop. Dis..

[46] Nunes D., Boa-Sorte N., Grassi M.F.R., Taylor G.P., Teixeira M.G., Barreto M.L., Dourado I., Galvão-Castro B. (2017). HTLV-1 is predominantly sexually transmitted in Salvador, the city with the highest HTLV-1 prevalence in Brazil. PLoS ONE.

[47] Carneiro-Proietti A.B.F., Amaranto-Damasio M.S., Leal-Horiguchi C.F., Bastos R.H.C., Seabra-Freitas G., Borowiak D.R., Ribeiro M.A., Proietti F.A., Ferreira A.S.D., Martins M.L. (2024). Mother-to-child transmission of human T-cell lymphotropic viruses-1/2: What we know, and what are the gaps in understanding and preventing this route of infection. J. Pediatr. Infect. Dis. Soc..

[48] Rosadas C., Taylor G.P. (2022). Current interventions to prevent HTLV-1 mother-to-child transmission and their effectiveness: A systematic review and meta-analysis. Microorganisms.

[49] Millen S., Thoma-Kress A.K. (2022). Milk transmission of HTLV-1 and the need for innovative prevention strategies. Front. Med..

